# A new nematicidal compound produced by *Streptomyces albogriseolus* HA10002

**DOI:** 10.1007/s10482-013-9890-8

**Published:** 2013-02-27

**Authors:** Qingfei Zeng, Huiqin Huang, Jun Zhu, Zhe Fang, Qianguang Sun, Shixiang Bao

**Affiliations:** 1 Guizhou Institute of Prataculture, Guiyang, People’s Republic of China; 2 Institute of Tropical Bioscience and Biotechnology, Key Laboratory of Tropical Crop Biotechnology, Ministry of Agriculture, Chinese Academy of Tropical Agricultural Sciences, Haikou, People’s Republic of China

**Keywords:** Nematicidal activity, *Streptomyces albogriseolus*, Spectroscopic analyses, Bioactive compound

## Abstract

Strain HA10002 was isolated from mangrove sediment collected from Dongzhaigang Mangrove Reserve in Hainan, China. It was selected with potent nematicidal activity and was identified as *Streptomyces albogriseolus*. By bioassay-guided fractionation, a new active component A22-1(S1) against root-knot nematodes was separated from its fermentation broth. On the basis of spectroscopic analyses and comparison with the data from correlative literature, the structure of S1 was established to be 6′-methyl-fungichromin, named as fungichromin B in this paper. The LD50 values of fungichromin B to the 2-stage juveniles of *Meloidogyne incognita* and *Meloidogyne javanica* were 7.64 and 7.83 μg/ml, respectively. Further examination demonstrated fungichromin B still showed a wide antifungal spectrum, as with fungichromin.

## Introduction

Root-knot nematodes (*Meloidogyne* spp.) are one of the three most economically damaging genera of plant-parasitic nematodes on horticultural and field crops, which destroy the root system of plants by penetrating into the roots. This soil-borne disease usually results in poor growth (Trudgill and Blok 2001), a decline in quality and yield of the crop and reduced resistance to other stresses. A high level of root-knot nematode damage can even lead to total crop loss (Sasser 1980; Sasser 1983). The measures taken to protect against root-knot nematodes are now dependent mainly on chemical nematicides, but such chemicals can also bring on health and environmental problems associated with their production and use (Thomason, 1987), so it is very important and necessary to take into consideration the biological method in controlling nematodes.

Of the biological control agents, actinomycetes are one (Oka et al. 2000) of the most important microbial resources, some of them produce metabolic substances which can inhibit (DeBoer and Dietz 1976; Capon et al. 2000; Ayers et al. 2007; Wang et al. 2011) or even kill nematodes, and developing such bioactive products is a promising (Sultana et al. 2010; Wang et al. 2010; Begum et al. 2011) field in biocontrolling *Meloidogyne* spp. (Oka et al. 2012). A few commercial products based on actinomycetes have been developed, but all so far have had only limited success. New bioactive compounds are needed for the treatment of root-knot nematodes, and new antibiotics may have novel mechanisms of diseases suppression, so the discovery of novel active substances for the control of *Meloidogyne* spp. appears to be very significant.

In the course of a program for bioactive compounds against root-knot nematodes, an actinomycete (designated as strain HA10002) was isolated from mangrove sediment samples collected from Dongzhaigang Mangrove Reserve, in Hainan Province, China. It exhibited distinct activities against *Meloidogyne* *incognita*, as well as *M. javanica*. This study focused on isolation, screening and identification of strain HA10002, separation and structure elucidation of the active constituents.

## Materials and methods

### Sample collection

Marine sediments were collected from Dongzhaigang Mangrove Reserve, Hainan Province, China, during the summer of 2010. Mangrove samples were conserved in ice-box and preprocessed within 12 h.

### Media and *Meloidogyne* spp.

The Gause inorganic agar (Gause et al. 1983) supplemented with a final concentration of 50 μg ml^−1^ potassium dichromate (K_2_Cr_2_O_7_) was used for actinomycete isolation, dissolved with 50 % old seawater at pH 7.2–7.4. Fermentation medium for actinomycetes was composed of 0.5 % glucose, 1.5 % soluble starch, 1.5 % yeast extract, 2.0 % bacterial peptone, 0.05 % K_2_HPO_4_, 50 % seawater, and pH 7.2–7.4.

The target nematodes for selecting actinomycetes against *Meloidogyne* spp., *M. incognita* and *M. javanica*, were kindly provided by Hainan Institute of Agricultural Environment and Plant Protection, Haikou, China.

### Isolation of actinomycetes

The sea mud samples were made ten-fold serial dilutions and coated in triplicate onto the Gause agar plates. Plates were incubated at 28 °C. When a mixture of colonies came into being on plates, each colony was transferred to another new Gause inorganic agar plate by means of streak-plate, until purified colonies were acquired. Pure actinomycete strains were kept temporarily on Gause inorganic slants and preserved in a glycerol suspension (20 %, w/v) at −70 °C for permanent preservation.

### Screening of actinomycetes against root-knot nematodes

For detection of nematicidal activities, each strain was cultured in a liquid fermentation medium under orbital shaking at 180 rpm for 6 d at 28 °C. Thereupon fermentation broth was centrifuged and the supernatant was tested for nematicidal activity using 24-well cell culture plate.

### Identification of actinomycete

Strain HA10002 was identified by morphology, cultural characteristics, physiological and biochemical properties, 16S rRNA gene and phylogenetic analysis.

### Purification of nematicidal compounds

The fermentation broth of strain HA10002 was extracted with ethyl acetate (EtOAc) at pH 7.5, and the extract was evaporated in reduced pressure to form a raw material. Afterwards the raw extract was chromatographed in silica gel column adopting the solvent system cyclohexane/acetone (6:1–3:1–1:1). The active fraction was separated via preparative TLC (chloroform/acetone, 4:1). Ultimately the component was purified through Sephedex LH-20 eluted with methanol to yield active compound A22-1 (S1).

### Structure identification of nematicidal active compounds

IR Spectra were recorded with a FT-IR Magna550 IR Spectrometer. NMR spectra were run in CDCl_3_ on Brucker DRX-500 instrument with TMS as internal standard. ESI-MS were determined applying a Mariner API-TOF instrument.

### Antifungal activity determination

Antifungal activity of new compound was measured by disk assay method (Ding et al. 2008). The test fungi for antimicrobial activity included *Saccharomyces cerevisiae*, *Fusarium oxysporum* and *Aspergillus niger*. They were inoculated on PDA medium plates, filter paper disks impregnated with methanol solution of the compound were placed on plates, using filter paper disks absorbed with methanol solution as control. When cultured at 28 °C for 3 to 5 days, sizes of fungistatic rings were observed.

## Results and discussion

A total of 216 actinomycete strains were isolated from 19 mangrove sediments samples, 6 antagonistic strains against *Meloidogyne* spp. were screened, of which the fermentation broth of strain HA10002 possessed prominent bioactivity and this strain also revealed stable genetic properties, and so it was selected to be further researched.

Through morphological observation, 16S rDNA and phylogenetic analysis, strain HA10002 was identified as *Streptomyces albogriseolus*, whose 16S rDNA GenBank accession number was HQ171094.

The nematicidal active compound A22-1(S1) produced by strain HA10002 was yellow amorphous powder, soluble in acetone, chloroform, DMSO, methanol, ethanol and insoluble in water and petroleum ether, its melting point was between 195 to 196 °C and modified bismuth potassium iodide reagent on TLC showed negative.

Ultraviolet spectrum revealed that compound S1 had maximum absorption at 320, 337 and 340 nm, suggesting a big conjugated system existed in its structure. Infrared spectrum showed the presence of hydroxyls (3,415, 1,067, 1,005 cm^−1^), ester bonds (1,722, 1,173, 1,139 cm^−1^), conjugated double bonds (3,022, 1,637, 847 cm^−1^), more than 4 contiguous methylene units (720 cm^−1^). ^13^C NMR and DEPT spectra (Table 1) showed a total of 36 carbon signals, of which there were three methyls carbons, ten methylene carbons, twenty-one methyne carbons (including ten olefinic carbons), two quaternary carbons (containing an ester carbon and an alkene carbon). FAB-MS spectra displayed [M+Na]^+^m/z 707, [M+H]^+^m/z 685, indicating the molecular weight of compound S1 was 684.

**Table 1:** ^13^C NMR data of compound S1 and Fungichromin (δ in ppm)

Position	S1	Fungichromin	Position	S1	Fungichromin
	*δ*_C_^a^	*δ*_C_^b^		*δ*_C_^a^	*δ*_C_^b^
1	172.4 (s)	173.0 (s)	19	136.2 (d)	135.4 (d)
2	60.2 (d)	60.3 (d)	20	133.4 (d)	134.1 (d)
3	73.6 (d)	73.7 (d)	21	133.7 (d)	134.8 (d)
4	41.2 (t)	41.4 (t)	22	132.9 (d)	133.7 (d)
5	74.2 (d)	74.1 (d)	23	133.6 (d)	134.2 (d)
6	44.4 (t)	45.2 (t)	24	130.3 (d)	132.0 (d)
7	73.8 (d)	73.9 (d)	25	133.9 (d)	134.3 (d)
8	44.9 (t)	45.3 (t)	26	72.6 (s)	73.2 (s)
9	74.2 (d)	74.2 (d)	27	74.9 (d)	75.2 (d)
10	44.1 (t)	44.3 (t)	28	18.3 (q)	18.0 (q)
11	71.3 (d)	71.5 (d)	29	12.3 (q)	11.7 (q)
12	40.8 (t)	39.6 (t)	1′	72.2 (d)	72.6 (d)
13	69.8 (d)	70.3 (d)	2′	36.1 (t)	36.2 (t)
14	78.3 (d)	78.3 (d)	3′	25.6 (t)	26.0 (t)
15	79.9 (d))	80.4 (d)	4′	30.1 (t)	32.9 (t)
16	140.1 (s)	138.6 (s)	5′	32.3 (t)	23.7 (t)
17	129.0 (d)	129.9 (d)	6′	23.1 (t)	14.4 (q)
18	128.3 (d)	129.1 (d)	7′	14.2 (q)	

^a^Recorded at 100 MHz in C_5_D_5_N

^b^Recorded at 100 MHz in MeOH-*d*_*4*_

**Fig. 1:**
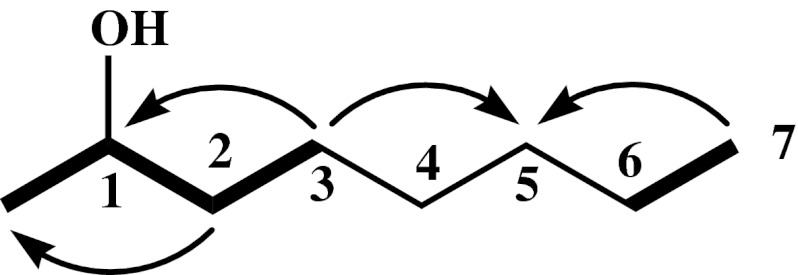
^1^H–^1^H COSY and HMBC of the side chain of compound S1

Searching online by Scifinder scholar the compounds whose molecular weight are 684 and the homologues with differences of one to three methylenes, and comparing with the data of the UV, IR and NMR spectra above, we inferred that compound S1 might well be the analogue of fungichromin. Comparing the data of the ^13^C, ^1^H NMR spectra of compound S1 with that of reported fungichromin (Noguchi et al. 1988), combining with analyzing the characteristics of HSQC, ^1^H–^1^H COSY, HMBC and ROSEY spectra of compound S1, we found that compound S1 harboured one more CH_2_ than fungichromin, and they possessed the same macrocyclic lactone. The difference of *δ*_C_ in ^13^C NMR spectra between compound S1 and fungichromin mainly lay in the methylenes from 10 to 36 ppm, and by means of the HSQC, ^1^H-^1^H COSY and HMBC spectra of compound S1, the data of this section were elucidated as follows: the ^13^C NMR spectra of compound S1 gave the information that there existed eight carbons whose chemical shifts were from 10 to 35 ppm, of which three were methyl carbons [*δ*_C_ 18.3 (C-28), *δ*_C_ 14.2 (C-7′), *δ*_C_ 12.3 (C-29)] and five were methylene carbons [*δ*_C_ 36.1 (C-2′), *δ*_C_ 25.6 (C-3′), *δ*_C_ 30.1 (C-4′), *δ*_C_ 32.3 (C-5′), *δ*_C_ 23.1 (C-6′)], thereof, C-28 and C-29 belonged to the lactone ring and the rest belonged to the methyl and methylene at side chain. ^1^H–^1^H COSY and HMBC spectra showed these carbons were in coupling relationships as shown in Fig. 1, and referring to the infrared spectra, it was revealed that there were at least four connected methylenes at the side chain. Thus, based on the data of ^13^C NMR, DEPT and FAB-MS spectra, the structure of compound S1 was determined to be 6′-methyl-fungichromin, we gave it the nomenclature “fungichromin B” in this paper.

**Fig. 2:**
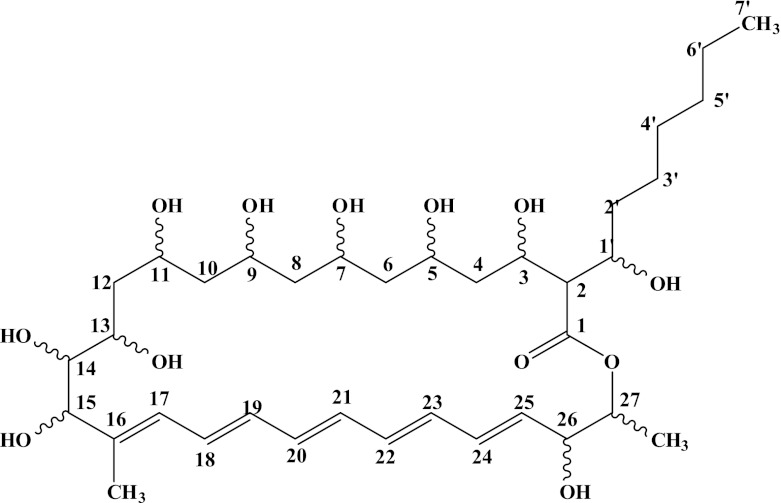
The Structure of fungichromin B

According to the carbon signals of ^13^C NMR and DEPT spectra of fungichromin B, comparison of the data of ^13^C, ^1^H NMR spectra of fungichromin B with that of reported fungichromin, and the molecular formula of fungichromin B displayed by FAB-MS spectra, the molecular formula of fungichromin B was determined to be C_36_H_60_O_12_, with the structure to be shown in Fig. 2.

Nematicidal activity of fungichromin B against *M. incognita* and *M. javanica* was measured by 24-well cell culture plate. According to the results of three repeated experiments, it was determined that the LD50 values of fungichromin B to the 2-stage juveniles of *M. incognita* and *M. javanica* were 7.64 and 7.83 μg/ml, respectively.

Fungichromin B belongs to the derivant of fungichromin, and fungichromin is the conjugate pentaene macrolide antibiotic produced by *Streptomyces cellulosae*, which exhibits a wide antifungal spectrum, resistant to yeast-like and filamentous fungi, and also shows toxic to mice (Noguchi et al. 1988). In order to find out whether fungichromin B also possessed antifungal activity, we carried out an antagonistic experiment specifically. The inhibition zones against three test fungi at ten-fold serial dilution concentrations were summarized in Table 2. The results showed that fungichromin B could antagonize yeast-like and filamentous fungi, demonstrating that in addition to nematicidal activity, fungichromin B still revealed a wide antifungal spectrum. In this work, we were aiming at searching new nematicidal compounds, and in view of the little antibacterial property of fungichromin, we did not further examine whether fungichromin B possessed the antibacterial activities.

**Table 2:** Inhibition zones of fungichromin B against three test fungi (diameter in mm)

Test fungi	Concentrations of fungichromin B		
	10.0 μg/6 mm disk	1.0 μg/6 mm disk	0.10 μg/6 mm disk
*Saccharomyces cerevisiae*	22	18	14
*Fusarium oxysporum*	19	15	12
*Aspergillus niger*	21	19	15

The values are the average of three experiments

## Conclusions

^1^H and ^13^C NMR data of fungichromin B (6′-methyl-fungichromin) and its in vitro nematicidal activity are demonstrated for the first time in this study. Experiments demonstrated that fungichromin B exhibited excellent activities against 2-stage juveniles of *M. incognita* and *M. javanica*. It could be a promising candidate as a natural microorganism-based product with nematocidal activity. In addition, this new compound was tested with antagonistic effect on yeast-like and filamentous fungi. In the following research, we will try to elucidate the inhibition mechanism of fungichromin B, and a wide range of *Meloidogyne* spp. will be examined.
